# “How many likes?”: The use of social media, body image insatisfaction and disordered eating

**DOI:** 10.1192/j.eurpsy.2021.1847

**Published:** 2021-08-13

**Authors:** I. Dias, J. Hernâni-Eusébio, R. Silva

**Affiliations:** Usf Do Minho, Agrupamento de Centros de Saúde Cávado I, Braga, Portugal

**Keywords:** social media, disordered eating, body image insatisfaction

## Abstract

**Introduction:**

Social media use has grown exponentially over the past few years, having a key role in communication among our youngsters. The impact of social media in mental health is still unclear. While some studies advocate that it increases social support and general satisfaction, others associate social media with the development of mental health issues. Social media can also perform some pressure regarding body image and eating behavior, caused by the idealistic appearance shown there in a very visual way.

**Objectives:**

This review intends to identify the existing evidence regarding social media use, its impact on body image and eating behavior.

**Methods:**

Search performed on May 11, 2020, including articles published since January 1st, 2006, written in Portuguese, English, Spanish and French. We used the MeSH terms ‘Body dissatisfaction’, ‘Body image’, ‘Feeding and eating disorders’, ‘Eating behaviors’ and ‘Social media’. The quality and strength of recommendation of the articles were evaluated using the Strength of Recommendation Taxonomy (SORT) scale from the American Academy of Family Physicians.

**Results:**

716 articles were initially found. Eight were selected: two systematic reviews, three cohort studies, two cross-sectional studies and one observational study.

**Conclusions:**

This review presents studies that establish a correlation between social media use, body image dissatisfaction and disordered eating. However, methodological and population heterogeneity can compromise the conclusions observed. With the current evidence, we can conclude that there is a relationship between the use of social media and changes in body image and/or eating behaviors in adolescents and young adults (SORT B).

**Disclosure:**

No significant relationships.

